# Paternal Age Explains a Major Portion of *De Novo* Germline Mutation Rate Variability in Healthy Individuals

**DOI:** 10.1371/journal.pone.0164212

**Published:** 2016-10-10

**Authors:** Simon L. Girard, Cynthia V. Bourassa, Louis-Philippe Lemieux Perreault, Marc-André Legault, Amina Barhdadi, Amirthagowri Ambalavanan, Mara Brendgen, Frank Vitaro, Anne Noreau, Ginette Dionne, Richard E. Tremblay, Patrick A. Dion, Michel Boivin, Marie-Pierre Dubé, Guy A. Rouleau

**Affiliations:** 1 Département des sciences fondamentales, Université du Québec à Chicoutimi, Saguenay, Canada; 2 Montreal Neurological Institute, McGill University, Montreal, Canada; 3 Centre de Pharmacogénomique Beaulieu-Saucier, Institut de Cardiologie de Montréal, Montreal, Quebec, Canada et Faculté de Médecine, Université de Montréal, Montréal, Canada; 4 Département de Psychologie, Université du Québec à Montréal, Montreal, Canada; 5 Département de Psychologie de l’éducation, Université de Montréal, Montreal, Canada; 6 École de Psychologie, Université Laval, Quebec, Canada; 7 School of Public Health, University College of Dublin, Dublin, Ireland; 8 Pediatrics and Psychology, University of Montreal, Montréal, Canada; Odense University Hospital, DENMARK

## Abstract

*De novo* mutations (DNM) are an important source of rare variants and are increasingly being linked to the development of many diseases. Recently, the paternal age effect has been the focus of a number of studies that attempt to explain the observation that increasing paternal age increases the risk for a number of diseases. Using disease-free familial quartets we show that there is a strong positive correlation between paternal age and germline DNM in healthy subjects. We also observed that germline CNVs do not follow the same trend, suggesting a different mechanism. Finally, we observed that DNM were not evenly distributed across the genome, which adds support to the existence of DNM hotspots.

## Introduction

Untill recently, little was known about the global prevalence of *de novo* mutations (DNM), and the factors that influence their rate of occurence. Some studies suggested that different factors such as pollution, tobacco smoke or magnetic field could impact the global mutation rate [1–3], but it was not possible to assess these on a genome-wide scale, or to look at different classes of DNM. New sequencing technologies now allow the interrogation of the full genome by sequencing. Using these technologies, it was recently demonstrated that paternal age effect (PAE) modulates the DNM rate in patients with psychiatric disorders[4, 5]. Here, we replicate these finding in ten disease free twin quartets. We show that rate of germline *de novo* single nucleotide variants (SNV) and indels but not CNV are associated with parental age. We also confirm the existence of DNM hotspots, suggesting new mechanisms for the occurrence of new mutations.

## Methods

### Recruitment

All patients gave informed consent in written form to participate in the Quebec Study of Newborn Twins. Ethic boards from the Centre de Recherche du CHUM, from the Université Laval and from the Montreal Neurological Institute approved this study. Amongst the cohort of the QSNT, we selected five families with young father at conception (range from 20–25) and five families with older father at conception (range from 40–47). The cohort did not allow us to select families with similar maternal age at conception for the two groups (range of 19–25 for the young father group and range of 30–36 for the older father group) (Table 1).

**Table 1:** Summary of DNM found for each twin quartet.

Group	Family ID	Paternal Age	Maternal Age	Twins	Base pair with coverage >20x (in Gbp)	DNM germline	FP Rate	Global germline DNM Rate*
Group 1								
	**TQ1**	45.27	30.15	Twin1	2,779	142	16,67%	2,14E-08
	** **			Twin2	2,776			
	**TQ2**	47.15	30.36	Twin1	2,779	96	10,99%	1,54E-08
				Twin2	2,779			
	**TQ3**	41.42	31.99	Twin1	2,795	91	14,29%	1,41E-08
	** **			Twin2	2,789			
	**TQ4**	40.8	36.77	Twin1	2,779	117	20,00%	1,69E-08
				Twin2	2,779			
	**TQ5**	42	34.68	Twin1	2,779	121	10,99%	1,94E-08
	** **			Twin2	2,776			
	** **					** **	**old father global DNM rate**	**1,74E-08**
Group 2	** **							
	**TQ6**	25.8	26.5	Twin1	2,795	60	10,00%	9,75E-09
	** **			Twin2	2,795			
	**TQ7**	24.8	19.91	Twin1	2,795	71	15,38%	1,08E-08
				Twin2	2,792			
	**TQ8**	23.55	21.81	Twin1	2,779	77	10,99%	1,24E-08
	** **			Twin2	2,779			
	**TQ9**	20.73	21.56	Twin1	2,779	58	10,99%	9,32E-09
				Twin2	2,789			
	**TQ10**	21.96	19.98	Twin1	2,795	56	23,08%	7,78E-09
	** **			Twin2	2,792			
						** **	**young father global DNM rate**	**1,00E-08**

*The Global germline DNM rate was corrected with the appropriated FP value for each family

### Sequencing

DNA from the 40 individuals was extracted directly from blood provided by the QSNT. Libraries were constructed using standard Illumina protocols. Sequencing was done on an Illumina HiSeq 2500 at Illumina sequencing facilities. Paired-end mode was used and the median fragment length was found to be ~300 bp for each individuals. Sequencing was performed until every sample reached an average coverage of 30x. For DNM rate calculation, only genome position with sufficient coverage (>20x) in both twins were kept for analysis (Table 1).

### SNP Genotyping

Genotyping was done using the Illumina HumanOmni2.5 genotyping arrays.

### CNV Genotyping

Log R Ratio (LRR) and B Allele Frequencies (BAF) were extracted by GenomeStudio (version 2011.1) from Illumina HumanOmni2.5 BeadChips using default parameters. CNVs were identified using QuantiSNP (version 2.3)[6] with local GC correction (hg19) and default parameters. For *de novo* mutations, putative CNV regions were created using the twins’ calls of any length but with a logarithm of the Bayes factor higher than 10. Only regions with higher than 25% reciprocal overlap found in both the twins were kept for further analysis. From this set, regions showing any sign of presence in one of the two parents (raw calls) or with an overlap with any regions found in the Database of Genomic Variants (DGV, version 10, November 2010) were discarded.

### SNV and indels calling

We received sequences from Illumina in the form of alignment (bam) files and variant (vcf) files. In order to have consistent metrics we applied our own bioinformatics pipeline and used the same genome reference (GRCh37), so we can compare with previous runs. We extracted the reads and created pools of 20M reads using Picard SAMtoFastq in order to optimise the use of our computing farm. Each of these pools were aligned using a modified version of the Burrows-Wheeler Aligner (bwa version 0.6.2-r126-tpx)[7] with threading enabled). The options were 'bwa aln -t 12 -q 5' and 'bwa sampe -t 12'. Next we recombined the pools in a single alignment (bam) file using Picard MergeSamFiles. The realignment method took roughly 36 hours walltime to complete.

We used the realigned bam file in GATK 1.6[8] to process the data with indel realignments, read duplicates removal and quality score recalibration. We made use of the scatter-gather mechanism in GATK to accelerate the process; walltime for these operations was around 40 hours. Once all 40 samples were processed, we generated QC metrics using GATK DepthOfCoverage and custom scripts (https://bitbucket.org/mugqic/mugqic_pipelines).

We used GATK UnifiedGenotyper to call SNVs and Indels in a run containing all samples, and splitting by chromosome to accelerate the process on the cluster. Finally, the variants were filtered and ordered using GATK Variant Recalibration, which assigns a validity score to each variant using a training algorithm. For this purpose we strictly followed the procedure from the Broad Institute (https://www.broadinstitute.org/gatk/guide/best-practices.php).

Once the variant calls were produced, these were compared to the SNP-chip calls; all samples had a variant concordance higher than 99.5%.

### WGS identification of SV

In order to achieve high-confidence structural variants calls for our samples, two different algorithms were used. The first one, *CNVer* v0.8.1[9], uses information from the paired-end mappings and from the depth of coverage in a given region to call CNVs (insertions and deletions). Prior to the mapping, the *FastqMcf* v1.1.2 (https://code.google.com/p/ea-utils/wiki/FastqMcf) utility was used to detect and remove fragments from sequencing adapters and primers and to remove poor quality bases at the end of reads. The *Bowtie* v0.12.9[10] aligner was then used with the “-v -2 –a–m 600—best—strata” options, as specified by the *CNVer* authors. To reduce the number of false positives, CNVs called by *CNVer* were merged when the distance to their neighbor was fewer than 5 kilobases.

The second algorithm, *BreakDancer[11]*, uses paired-end mappings signatures to identify different types of structural variants, including insertions, deletions, inversions and translocations. The reads were processed as described in the *SNV and indels calling* section before using *BreakDancerMax* (cpp implementation, version 1.1.2) to call structural variants. Once again, CNVs were merged with a 5 kilobase proximity threshold.

The germline DNM detection and exclusion from this dataset required a maximum overlap between every twin and his parents of 1% and a minimum overlap to the other twin of 90%.

### Sanger sequencing validation

Primers were designed using in-house scripts and the Batch Primer 3 web tool (http://probes.pw.usda.gov/batchprimer3/) using enough flanking sequence to have a good coverage of selected variants. PCR was performed using the AmpliTaq Gold DNA Polymerase (Applied Biosystems) according to the manufacturer’s instructions. To visualize DNA fragments, a small fraction of the PCR product was loaded on a 1.5% agarose gel containing ethidium bromide. PCR products were sequenced at the Genome Quebec Innovation Centre using a 3730XL DNAnalyzer (Applied Biosystems), and SeqMan from the DNAstar suite was used for mutation detection analysis.

## Results

In order to evaluate the effect of paternal age on the rate of DNM in healthy subjects, we selected ten families consisting of two monozygotic twins and their parents. All individuals were recruited by the Quebec Study of Newborn Twins (QSNT) project[12]. These families have been prospectively followed since birth and no severe health conditions were reported for the twins, now all around 15 years old. These families were selected according to extremes in paternal age at the time of conception. A group of 5 younger-aged fathers (mean = 23.37±SD 2.06) and 5 older-aged fathers (mean = 43.33±SD2.75) (Table 1) were sequenced. Paternal age at conception was significantly different between the two groups (t-test, p-value < 1.00∙10^−5^). Despite our efforts to select comparably aged mothers, there was also a small but statistically significant difference in maternal age at conception (t-test, p-value < 0.0065). Thus, we will be able to directly assess the effect of parental age effect on DNM variability and we will need to establish parental origin of mutations to directly implicate the paternal or maternal age.

Whole Genome Sequencing (WGS) was performed on all 40 family members from the 10 families. Mutation detection was performed using an in-house bioinformatics pipeline to optimize the detection of variants present in the twins against the variant profiles of the parents. All samples were genotyped using Illumina HumanOmni2.5 BeadChips and the SNP calls were used to calibrate and validate variant detection with the WGS bioinformatics pipeline. *de novo* CNVs were identified using data from the Illumina SNP-chips as well as from the WGS data using two different algorithms[9, 11]. Consistent DNMs in a twin pair were categorized as germline (i.e. inherited from gamete cells of one of the parents), while DNM found in a single twin were categorized as post-zygotic (i.e. mutations happening post-conception).

Overall, 889 germline mutations and 106 post-zygotic mutations were detected using these criteria in the 10 twin pairs (S1 Table). In order to test the validity of our *in silico* detection pipeline, we tested a subset of the DNM identified using Sanger Sequencing. The proportion of SVs and indels validated was based on the total proportion observed in the full dataset. A total of 91 germline variants as well as 62 post-zygotic variants were tested using Sanger sequencing in the corresponding quartet. Almost 88% of the germline SNV variants were validated while the indels validation rate was slightly better at 93.5%. Based on these results, we calculated a false positive rate for each quartet and we used these individual rates to correct the total number of DNM. For family quartets for which no false positive was identified, we corrected with the overall false positive rate of 10.9%. For the post-zygotic variants, only 9 of 62 variants were confirmed, resulting in a false positive rate higher than 85%. Thus, given that we would predict that we have detected only 15 post-zygotic DNM, it is impossible to arrive at any conclusions regarding post-zygotic DNM rates, except to say that they are in limited number when looking at DNA extracted from blood.

Assuming a single mutation event for all SNV and indels germline mutations and collapsing all twin pairs to a single individual, we calculated the germline *de novo* mutation rate to be 1.37∙10^−8^, which is higher than the previously reported rate of 1.1∙10^−8^.[13]. However, when stratified by paternal age, the estimated DNM rate in the groups of younger and older fathers respectively were 1.01∙10^−8^ and 1.74∙10^−8^ (Rao-Scott chi-square p = 1.38∙10^−12^)(Table 1). We then looked at the CNV DNM rates. CNVs were identified using three different methods, one using the SNP genotyping arrays and two using algorithms that predict CNVs from the, one focussing on the depth of coverage information (CNVer) and another that uses read pairs mapped with unexpected distance or orientation (BreakDancer). For the three algorithms, we kept only CNVs that had an overlap >90% in the two twins and no overlap at all with the parents. As getting a consensus for CNV is not trivial and was not needed here, we tested for parental age effect on each separate dataset without building a consensus dataset. The results were all concordant and no association with parental age was seen for any method (Table 2)

**Table 2:** CNVs identified for each family.

Group	Family ID	Paternal Age	Maternal Age	QuantiSNP Loss	QuantiSNP Gain	CNVer Gain	CNVer Loss	BreakDancer Loss*
**Group 1**								
	**TQ1**	45,27	30.15	8	6	1	10	3
	**TQ2**	47,15	30.36	7	4	4	6	4
	**TQ3**	41,42	31.99	2	3	4	9	5
	**TQ4**	40,8	36.77	1	3	3	6	7
	**TQ5**	42	34.68	0	1	5	6	5
**Group 2**								
	**TQ6**	25,8	26.5	2	0	5	7	4
	**TQ7**	24,8	19.91	4	3	2	4	7
	**TQ8**	23,55	21.81	2	9	5	5	1
	**TQ9**	20,73	21.56	3	5	2	7	3
	**TQ10**	21,96	19.98	4	4	3	10	6

*Breakdancer was not able to confidently identify gain in our dataset

**Fig 1:**
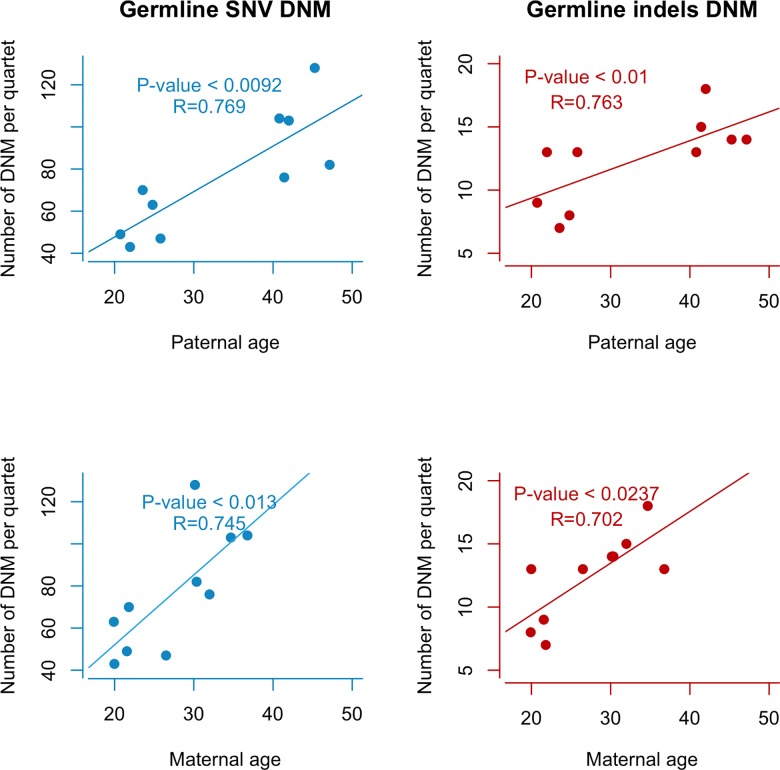
Association of parental age with germline DNM. This figure shows the correlation between different SNV and indel germline DNM (left: germline SNV, right: germline indels) with parental age (Top: paternal age, bottom: maternal age). The X-axis represents the parental age at conception. The Y-axis represents the number of DNM mutations identified through Whole Genome Sequencing.

**Fig 2:**
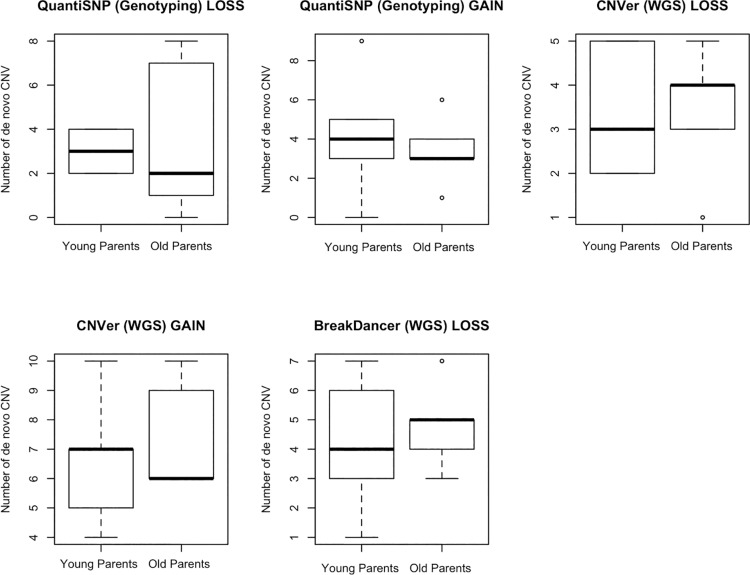
Association of parental age with CNV called by different algorithms. We used three different algorithms to detect CNV in our dataset. QuantiSNP was used for genotyping assays while CNVer and BreakDancer were used for WGS. Although the number varies according to which algorithm was used, no difference between young parental age group and old parental group can be detected.

We next sought to find if there was a specific association between parental sex and the variability in DNM rate. We found that there was a strong correlation between the germline DNM and paternal age (Poisson regression R^2^ = 0.78, p = 0.00066) as well as with maternal age (Poisson regression R^2^ = 0.65, p = 0.004), though the effect size is smaller for the later. Then, we tested for parental effect looking separately at single nucleotide variants (SNV) and small insertions and deletions (indels) (Fig 1). We found a strong correlation between paternal age and single nucleotide germline DNM variants (Spearman correlation R = 0.7695, p = 0.0092). This correlation was also observed in the mother, although not as strong (Spearman correlation R = 0.745, p = 0.013) suggesting that parental age accounts for an important portion of the variability in the DNM rate. A positive correlation was also found for indel germline variants for both paternal (Spearman correlation R = 0.7631, p = 0.01) and maternal age (Spearman correlation R = 0.702, p = 0.0237). The weaker association with indels could in part result from the lower power due to the comparatively less frequent occurrence. This observation is nonetheless in concordance with other studies[4, 5]. Interestingly, germline *de novo* CNVs show no association with parental age (Fig 2) (Student T test > 0.05 for all five comparisons). Although further validation is required, this would suggest that the mechanisms explaining the increasing rate of SNV DNM with increasing parental age do not apply to CNVs, which may have implications in our understanding of this phenomenon.

In order to separate out the paternal and maternal age effects, we used individual WGS reads to assign parental origin of the germline DNM. Working only with germline SNVs, as they were more frequent than indels, we assigned parental origin according to co-transmission of DNM with polymorphisms on single sequence reads. Out of 765 DNM, we were able to trace parental origin for 57 DNM. Of those, 43 DNM were of paternal origin and 14 were of maternal origin; providing an estimated paternal:maternal ratio of ~ 3:1 in our dataset. When limiting the comparison to the younger paternal age group, we see 16 paternal germline DNM and 5 maternal germline DNM (ratio of ~ 3:1) whereas the older paternal group has 27 paternal germline DNM and 9 maternal germline DNM (ratio of ~ 3:1). These data suggest that DNM rates increase both with maternal and paternal age, though the paternal age explains a greater number of DNMs than maternal age.

## Discussion

Many groups have shown an association of *de novo* mutations (DNM) to neurodevelopmental disorders, notably intellectual disability (ID)[14], autism spectrum disorder (ASD)[15–17] and schizophrenia[18–20]. At the time these studies were published, it was yet unclear whether the DNM rate was fixed across our species or if it was subject to variation. Interestingly, it was previously shown that paternal age at conception is positively correlated with risk of schizophrenia[21, 22].

In this study, we show that parental age, more specifically paternal age, explains a major portion of the variation in the germline DNM rate. Contrary to other studies that have reported similar findings, we have studied disease free families, and we have isolated the germline DNM rate from post-zygotic mutations with respect to parental age[4, 5]. In our study, fathers aged 40 or greater had a rate of germline DNM that was almost twice as high as that of fathers aged twenty years younger. We also observe a smaller, yet significant increase in the mutation rate associated with maternal age. Although we were not able to fully account for age-dependent sex-effect because of assortative mating present in our families, the origin of mutation suggests that older maternal age also leads to an increase in DNM. This conclusion is also supported by other studies that also report a maternal age effect on DNM rate[23–25]. The rates that we observed in this study are comparable to the rates reported previously[4, 5]. These findings do not appear to apply to CNVs. We have observed that the CNV rate does not vary with parental age. This is not a surprising finding as it is known that CNV and SNV have different mechanism or origin although the technical variation observed specifically for CNV in this study does not permit to fully exclude the link between CNV and parental age. It is thought that most CNVs in the human genome arise from non-allelic homologous recombination while a vast majority of the SNVs occur during DNA replication[26]. The implications of these findings are important, as an increased DNM rate could impact a number of Mendelian and complex genetic disorders. A study design that includes the DNA of two parents and their children has proved a valuable approach for the investigation of DNM. The ability to assess DNM rates offers a valuable means to further characterize the impact of rare variants on diseases and traits and should be encouraged in the design of future studies where parents are available.

## Supporting Information

S1 Table List of all *de novo* mutations identified through WGS. (XLSX)
